# Atrial Fibrillation Ablation in Patients With Chronic Kidney Disease: A Review of Literature

**DOI:** 10.7759/cureus.46545

**Published:** 2023-10-05

**Authors:** Arjun Basnet, Azka Naeem, Nava R Sharma, Saral Lamichhane, Sajog Kansakar, Sudarshan Gautam, Kripa Tiwari, Armando Seitillari, Remil Thomas, Kalyana Janga

**Affiliations:** 1 Internal Medicine, Maimonides Medical Center, Brooklyn, USA; 2 Medicine, Manipal College of Medical Sciences, Pokhara, NPL; 3 Internal Medicine, Gandaki Medical College, Pokhara, NPL; 4 Internal Medicine, Manipal College of Medical Sciences, Pokhara, NPL; 5 Internal Medicine, Nuvance Health Vassar Brothers Medical Center, New York, USA; 6 Nephrology, Maimonides Medical Center, Brooklyn, USA

**Keywords:** atrial fibrillation ablation, atrial fib ablation, radio-frequency ablation, atrial fibrillation recurrence, chronic kidney disease (ckd)

## Abstract

Atrial fibrillation (AF) is a common arrhythmia among patients with chronic kidney disease (CKD), which leads to increased cardiovascular complications. Catheter ablation (CA) has emerged as an effective and safe treatment for AF in CKD patients. CA offers tailored treatment strategies and presents a safer alternative with fewer adverse outcomes than anti-arrhythmic agents. Although CKD patients undergoing ablation have similar complication rates to non-CKD patients, they face a higher risk of hospitalization due to heart failure. Furthermore, CA shows promise in improving kidney function, particularly in individuals who maintain sinus rhythm. Future research should address limitations by including advanced CKD patients, conducting longer-term follow-ups, and developing individualized treatment approaches.

## Introduction and background

Atrial fibrillation (AF) is the most common arrhythmia in patients with chronic kidney disease (CKD) [1]. AF is associated with increased cardiovascular complications within the CKD patient population, including stroke, heart failure, and mortality. CKD and AF share common risk factors, including diabetes, hypertension, and coronary artery disease. The global prevalence of CKD is estimated at 13.4%, and the global estimate of AF burden in 2010 was 33.5 million [2]. Studies have shown that the prevalence of AF increases with the worsening of the estimated glomerular filtration rate (eGFR), while AF also increases the risk of CKD [3]. More alarmingly, the prevalence of AF amongst patients suffering from end-stage renal disease (ESRD) undergoing dialysis skyrockets to an estimated 15-40% [1]. Most of the treatment guidelines for AF are derived from non-renal cohorts, potentially making them less applicable to the CKD population. Treatment involves pharmacological rate, rhythm control, and oral anticoagulants [4].

Given that most anti-arrhythmic drugs and anticoagulants are excreted via the kidney, they can lead to serious adverse events due to increased serum concentrations in CKD patients [5,6]. As a result, radiofrequency catheter ablation (RFCA) is being explored as a potential alternative therapy for hemodialysis and non-hemodialysis CKD patients. A handful of studies have demonstrated satisfactory long-term outcomes of AF post-RFCA in non-hemodialysis patients [7]. However, data regarding these outcomes in hemodialysis patients remain limited.

## Review

Relationship and pathophysiology of AF in CKD

Some studies show an essential relationship between AF and CKD; the incidence of AF increases significantly by 2- to 3-fold in CKD patients compared with the general population, while AF is strongly associated with the development and progression of CKD [8-11]. Further studies have demonstrated that AF may be one of the contributing factors in worsening CKD and potentially leading to ERSD requiring hemodialysis [12,13]. As this bidirectional relationship is important, there are significant limitations to it as well. It is well known that CKD is more prominent in older populations associated with coronary artery disease, heart failure, and hypertension. Hence, it is challenging to design studies that account for these potential confounders. 

The underlying mechanistic processes that connect AF with CKD are abundant. Both conditions are believed to have a strong link with inflammation. It has been elucidated that the inflammatory markers are elevated in CKD and play a crucial role in the disease process as it evolves [14]. Furthermore, it has been reported that inflammatory markers are essential in initiating and progressing AF [15]. Coronary artery disease is the main driving cause of AF in this population. Endothelial dysfunction and inflammation often seen in CKD patients lead to accelerated atherosclerosis and, in turn, promote initiation and persistence of AF [15-17].

Another important mechanistic process that connects AF and CKD is the renin-angiotensin-aldosterone system (RAAS). The pathogenesis of CKD is multifactorial, but RAAS plays a vital role by increasing the production of fibrotic growth factors, reactive oxygen species (ROS), and several other factors linked to the development of atrial fibrosis and enlargement [18-20]. Although the exact mechanism is unknown, fibrotic growth factors and oxidative stress through ROS and RAAS are involved in developing atrial fibrosis and enlargement. Several models with the transgenic mouse, which had angiotensin-converting enzyme (ACE) overexpressed, demonstrated preferential growth and fibrosis of the atria compared to the ventricle; consequently, the development of AF was more prominent [21]. In other clinical and animal trials, it has been reported that ACE inhibitors have a protective role in reducing atrial fibrosis and decreasing AF occurrence [22]. Furthermore, the buildup of toxins in CKD, such as the uremic toxin indoxyl sulfate, increases the calcium levels in atria and pulmonary veins, which plays a vital role in the AF disease process, such as triggering ectopic activity and initiating reentry phenomena [23].

**Figure 1:**
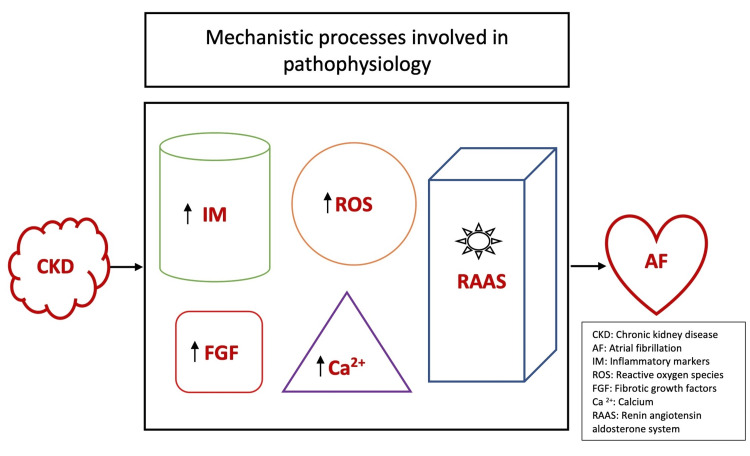
A schematic diagram of the underlying mechanistic processes involved in the pathophysiology of AF in CKD patients

Therefore, changes in calcium levels via the buildup of toxins, activation of RAAS, fibrotic growth factors, and oxidative stress via ROS are some of the several mechanisms that link the pathophysiology of CKD with AF, as illustrated in Figure 1.

AF ablation: an overview

Managing AF includes two significant objectives: alleviating symptoms (palpitations, dyspnea, and exercise intolerance) with rate or rhythm control and preventing thromboembolism. The first objective can be achieved with rate control agents, anti-arrhythmic drugs, electrical cardioversion, and ablation via a catheter or surgical approach. Catheter ablation (CA) for AF is an effective and safe approach to treat AF that is being increasingly performed worldwide and is one of the most common electrophysiology procedures performed today. The 2017 HRS/EHRA/ECAS/APHRS/SOLAECE expert consensus statement on catheter and surgical ablation of AF recommends CA in symptomatic AF refractory or intolerant to at least one Class I or III anti-arrhythmic medications [24]. The recommendation is class I for paroxysmal AF, class IIA for persistent AF, and class IIB for long-standing AF. 

Pulmonary vein isolation (PVI) is the mainstay AF ablation technique. The two most popular approaches to achieving pulmonary vein isolation are radiofrequency ablation (RFA) and cryoablation. Clinical trials have demonstrated both approaches to be similar in efficacy and safety [25-27]. Many advancements in RFA and cryoablation have been made recently to achieve more durable PVI by limiting PV reconnection. Wide-area circumferential ablation with conduction block verification via an antral approach is now recommended for all AF ablation procedures (class IA recommendation) [24]. Adjunctive ablation strategies are being evaluated, especially for persistent and long-standing AF, as they are more likely to involve mechanisms beyond pulmonary vein arrhythmogenesis due to substantial atrial remodeling. Many structures have been suggested as sites of non-pulmonary vein triggers, such as the posterior wall of the left atrium, the left atrial appendage (LAA), the superior vena cava, the crista terminalis, the fossa ovalis, the coronary sinus, the ligament of Marshall, and adjacent areas of atrioventricular valve annuli [28, 29]. However, no evidence exists for any adjunctive ablation strategy yet. 

The CABANA (Catheter Ablation Versus Antiarrhythmic Drug Therapy for Atrial Fibrillation) trial demonstrated that CA was effective in reducing the recurrence of AF by 48% and symptomatic AF by 51% compared with drug therapy over five years of follow-up [30]. For paroxysmal AF, studies have shown that the rate of freedom from recurrence can reach 60-79% [31]. Efficacy is lower for persistent and long-standing AF [32]. Complications from the procedure included cardiac tamponade, pulmonary vein stenosis, phrenic nerve paralysis, stroke, and atrial-esophageal fistula; however, periprocedural complication rates remained low [33]. Following CA, growing evidence supports the management of risk factors as an essential step to improve outcomes. BMI is an important factor in determining outcomes. In addition to diet and exercise, surgical weight loss improves outcomes after CA [34,35]. There may be a role for alcohol abstinence and exercise [36].

The 2017 consensus statement recommends anticoagulation for at least two months post-CA with warfarin or novel oral anticoagulants [24]. The decision to stop anticoagulation after two months should be based on the patient’s risk of stroke, i.e., CHA2DS2-VASc score. Optimal anticoagulation after successful ablation in a patient with high stroke risk remains unknown. There are no long-term clinical trials on the topic, but there are ongoing studies to identify the optimal timeframe [37].

Comparison of AF ablation with medical therapy in CKD patients: safety and effectiveness

Numerous studies have consistently demonstrated the association between CKD and unfavorable AF outcomes, summarized in Table 1.

**Table 1:** Summary of studies including ablation for atrial fibrillation in CKD patients. CKD: chronic kidney disease, eGFR: estimated glomerular filtration rate, AF: atrial fibrillation, TIA: transient ischemic attack, HR: hazard ratio, AF/AFL: atrial fibrillation/atrial flutter, ESRD: end-stage renal disease, HD: hemodialysis, CCr: creatinine clearance, LA: left atrium, LAV: left atrial volume.

Title of Article	Authors	Study Design	Target Population	Sample Size	Aim of Study	Follow-up Period	Results or Outcome	Limitations
Pharmacological cardioversion in patients with recent-onset atrial fibrillation and chronic kidney disease sub-analysis of the CANT II study	Ceynowa-Sielawko et al. [41], 2022	Sub-analysis of multicenter, retrospective registry-based CANT II trial	Patients with paroxysmal AF admitted for urgent restoration of sinus rhythm	1365	To evaluate the safety and effectiveness of pharmacologic cardioversion, in particular with intravenous antazoline in comparison to amiodarone, propafenone, and/or overlapping therapy (secondary aim) administered to patients with CKD with paroxysmal AF	Not available	In patients receiving amiodarone, the PCV success rate was similar in all the studied groups, but along with a renal function decline, it decreased in patients receiving antazoline (79.1 vs. 35%; p < 0.001), and it increased almost significantly in patients receiving propafenone (69.9 vs. 100%; p = 0.067) - Safety profile of antazoline remained unchanged with decreasing eGFR	-Highly heterogeneous patient population -Not enough population with advanced CKD
Efficacy and safety of dronedarone versus placebo in patients with atrial fibrillation stratified according to renal function: Post hoc analyses of the EURIDIS‐ADONIS trials.	Thind et al. [42], 2022	Post-hoc analysis of multicenter, double‐blind, parallel‐group trials	Patients with non-permanent AF have had at least one episode in the preceding three months	1229	To assess the dronedarone efficacy and safety for controlling sinus rhythm in patients with non-permanent AF	up to 12 months	The median time to first AF/AFL recurrence was significantly longer with dronedarone versus placebo for all eGFR subgroups except the 30 to 44 mL/min group (HR 0.620), where the trend was the small population may have limited similar but statistical power. Dronedarone may be a suitable treatment choice for patients with a 45 mL/min renal function or higher. The study showed that it did not require dose adjustment or ongoing monitoring of renal function for these patients	Continuous monitoring of AF was not performed. Creatinine levels were not systematically collected after the end of the EURIDIS‐ADONIS trial. Hence, data on the reversibility of elevated creatinine values are not available in this particular study population
Safety and clinical outcomes of catheter ablation of atrial fibrillation in patients with chronic kidney disease	Ullal et al. [7], 2016	Retrospective cohort study based on database	Patients with AF and at least one year of CKD who underwent catheter ablation	21,091	To evaluate the 30-day complications and 1-year outcome in patients with CKD after the first catheter ablation	30 days and one year	At 30 days post-ablation, patients with CKD had similar rates of stroke/TIA (0.13% vs. 0.13%, p = 0.99), perforation/tamponade (3.2% vs. 3.1%, p = 0.83), and vascular complications (2.4% vs. 2.2%, p = 0.59) as patients without CKD, but were more likely to be hospitalized for heart failure (2.1% vs. 0.4%, p < 0.001). No significant differences in hazards of AF hospitalization (adjusted HR: 1.02, 95%CI: 0.87–1.20), cardioversion (adjusted HR: 0.99, 95%CI: 0.87–1.12), or repeat AF ablation (adjusted HR: 0.89, 95%CI: 0.76–1.06) at one year	- Data predated the introduction of CPT codes specific to ablation for AF. - Lack of information on the severity of CKD or the use of anticoagulation therapy. - The study did not compare catheter ablation with medical management and had a short-term follow-up
Five-year change in renal function after catheter ablation of atrial fibrillation	Park et al. [44], 2019	Single-center observational cohort study	Patients who underwent AF catheter ablation and those with AF under medical therapy in the National Health Insurance Service database	16,019	Comparison of patients who underwent catheter ablation for AF with those with medical management	Five years	AF catheter ablation, but not medical therapy, improved eGFR considerably (p < 0.001). Improvement in long-term renal function was more significant in individuals who remained in sinus rhythm after the last AF ablation session and those who did not have preexisting diabetes mellitus	- Did not include patients with advanced CKD; hence, the results cannot be generalized. - Did not explore the underlying mechanisms of the association between renal function improvement and maintenance of sinus rhythm.
Impaired renal function is associated with recurrence after cryoballoon catheter ablation for paroxysmal atrial fibrillation: a potential effect of non-pulmonary vein foci.	Yanagisawa et al. [45], 2017	Retrospective cohort study based on database	Patients undergoing second-generation cryoballoon catheter ablation for paroxysmal AF	110	Association between estimated eGFR and outcomes after cryoballoon catheter ablation for AF	Nine months	Among the CKD groups, recurrence was found in 46% (of the eGFR eGFR 30-59.9 mL/min/1.73 m^2^ groups (p = 0.001) - Kaplan-Meier survival curves demonstrated that patients with eGFR 30-59.9 mL/min/1.73m^2^ had a significantly worse prognosis than the other groups (log-rank p < 0.001)	- Small sample size - Short follow-up duration - Lack of successful elimination of non-PV ectopic beats - Potential underestimation of AF recurrence due to monitoring tests
Catheter ablation for atrial fibrillation in patients with chronic kidney disease and on dialysis: a meta-analysis and review	Chung et al. [46], 2022	Metanalysis	Patients with AF have CKD or ESRD on HD		To assess the recurrence rate of AF after catheter ablation in patients with CKD or ESRD	25.5 months	Patients with CKD (RR 2.34, 95% CI: 1.36-4.02, p < 0.01)) and those on HD (RR 1.50, 95% CI: 0.84-2.67, p = 0.17) were more likely to experience AF recurrences following catheter ablation than otherwise healthy AF patients	- The study was composed of observational studies with differing protocols and heterogeneity in outcomes - Subgroup analysis stratified by CKD stage was not performed, as only a few studies were found - All the studies regarding catheter ablation in HD patients were performed in Japan, limiting study generalizability
Decreased estimated glomerular filtration rate predicts long-term recurrence after catheter ablation of atrial fibrillation in mild to moderate renal insufficiency	Zheng et al. [47], 2021	Single-center retrospective study	Patients with symptomatic AF despite using at least one antiarrhythmic drug or prior attempts of electrical cardioversion	306	To evaluate the predictors of the prognosis of catheter ablation for AF, especially the effect on renal function	27.2± 19.5 months	- The recurrence group had larger left atrial dimensions, higher LAV index, and lower kidney function (eGFR (p < 0.001) and CCr(< 0.05)). - Both eGFR and LAV indices were identified as independent factors associated with long-term recurrence after catheter ablation, indicating their prognostic significance	- Patients with severe renal insufficiency were excluded. Participants had a mean eGFR of mean eGFR 61.4 ± 14.8 mL/min/1.732 - Lacked comprehensive laboratory indicators to explore the underlying mechanisms (e.g., inflammatory markers, LA fibrosis) -Limited follow-up period

A post hoc analysis of the antithrombotic agent AF (ATA-AF) study revealed an independent link between impaired kidney function and a worse prognosis for AF patients, leading to increased cardiovascular morbidity and mortality [38]. Kovačević et al. found that AF and its recurrence were associated with a decline in kidney function, as indicated by a decreased eGFR. At the same time, patients free of arrhythmia experienced improved kidney function [39]. Thus, it is crucial to identify the most effective approach to managing AF in patients with CKD, aiming to minimize negative outcomes and recurrence rates.

In patients with CKD, rate control agents and anticoagulants are commonly used therapeutic agents, while a relatively small percentage undergoes CA or cardioversion compared to individuals without CKD [40]. Ceynowa-Sielawko et al. demonstrated that the effectiveness of different drugs varies in the presence of CKD. Propafenone and amiodarone maintained their known efficacy in terminating AF at lower eGFR. In contrast, although relatively safe, antazoline was less effective in patients with eGFR below 45 mL/min/1.73 m^2^ level [41]. However, this study had a highly heterogeneous patient population and lacked participants with advanced CKD. Another study by Thind et al. suggested that dronedarone could be a potential treatment option for patients with eGFR > 45 mL/min or higher, as it did not require dose adjustment or continuous monitoring of renal function [42].

Comparatively, CA has shown a better safety profile than antiarrhythmic agents due to CKD's potential impact on drug clearance and the increased risk of toxicity and adverse effects with antiarrhythmic medications. Kwazi et al. demonstrated that patients who underwent ablation had significantly lower rates of adverse outcomes, including all-cause death, cardiovascular death, hospitalization for heart failure, ischemic stroke, and major bleeding, compared to conservative management (14.7% versus 25.4% at eight years, log-rank p = 0.008) [43]. Ullal et al., in a retrospective cohort study, found comparable rates of postprocedural complications between CKD and non-CKD patients, indicating the safety of the procedure. At 30 days post-ablation, patients with CKD had similar rates of stroke/TIA (0.13% vs. 0.13%, p = 0.99), perforation/tamponade (3.2% vs. 3.1%, p = 0.83), and vascular complications (2.4% vs. 2.2%, p = 0.59) as patients without CKD but were more likely to be hospitalized for heart failure (2.1% vs. 0.4%, p < 0.001). However, this study's data predated the introduction of CPT codes specific for ablation for AF, lacked information on the severity of CKD or the use of anticoagulation therapy, and did not compare CA to medical management with short-term follow-up [7].

Regarding the effect of ablation on the improvement of glomerular filtration rate (GFR), a single-center observational study conducted by Park et al. observed a notable improvement in eGFR (p < 0.001) following CA. At the same time, medical therapy did not produce the same effect. Moreover, the improvement in long-term renal function was particularly significant in patients who maintained sinus rhythm after the final AF ablation session [44]. However, the generalizability of this study is limited since it did not include patients with advanced CKD and did not explore the underlying mechanisms of the association between renal function improvement and maintenance of sinus rhythm.

A study by Yanagisawa et al. aimed to explore the prognosis and recurrence rates following CA. They found that patients with an eGFR of 30-59.9 mL/min/1.73 m^2^ had a worse prognosis and higher chance of recurrence (46%, p < 0.001), which might be attributed to the lack of successful elimination of non-pulmonary vein ectopic beats. Therefore, further studies are warranted to explore the prognosis and recurrences following ablation [45]. Similarly, a meta-analysis conducted by Chung et al. in Japan supported these findings, showing that patients with CKD or ESRD on hemodialysis (HD) who underwent AF ablation experienced more AF recurrences than patients without kidney disease. Patients with CKD (RR 2.34, 95% CI: 1.36-4.02, p < 0.01) and those on HD (RR 1.50, 95% CI: 0.84-2.67, p = 0.17) were more likely to experience AF recurrences following CA than otherwise healthy AF patients. However, this meta-analysis had limitations, including its composition of observational studies with differing protocols, heterogeneity in outcomes, and lack of subgroup analysis stratified by CKD stages due to limited available studies. Additionally, the study focused on HD patients in Japan, limiting its generalizability [46]. Despite these limitations, both studies consistently demonstrated a poor prognosis and higher chances of recurrence in patients with CKD following AF ablation, suggesting further exploration.

A single-center retrospective study by Zheng et al. aimed to evaluate the predictors of the prognosis of CA for AF. They found that left atrial volume index (LAVI) and eGFR were independent factors associated with long-term AF recurrence following CA, highlighting their prognostic importance. The recurrence group had larger left atrial dimensions and higher LAV index (both p < 0.01) but lower kidney function (eGFR p < 0.001 and CCr p < 0.05). However, this study excluded patients with severe renal insufficiency, and the follow-up period was limited [47].

Hence, further research, including prospective randomized controlled trials, is needed to understand better the relationship between CKD and AF, better optimize treatment strategies, and improve long-term outcomes for patients with both conditions.

## Conclusions

In conclusion, additional prospective studies are warranted to comprehensively evaluate the effectiveness of ablation in managing AF among patients with CKD. The impact of a lower eGFR on prognosis and AF recurrence rates following ablation underscores the need for tailored treatment strategies. Nevertheless, CA presents a safer alternative with fewer adverse outcomes than antiarrhythmic agents. While CKD patients undergoing ablation exhibit similar complication rates, they are at a higher risk of hospitalization due to heart failure. Notably, ablation holds promise for improving kidney function, particularly in individuals maintaining sinus rhythm. Future research should address limitations such as including advanced CKD patients and longer-term follow-up, developing individualized treatment approaches, and promoting collaborative efforts between cardiologists and nephrologists to optimize AF management in CKD patients.
